# The Role of Vector Trait Variation in Vector-Borne Disease Dynamics

**DOI:** 10.3389/fevo.2020.00189

**Published:** 2020-07-10

**Authors:** Lauren J. Cator, Leah R. Johnson, Erin A. Mordecai, Fadoua El Moustaid, Thomas R. C. Smallwood, Shannon L. LaDeau, Michael A. Johansson, Peter J. Hudson, Michael Boots, Matthew B. Thomas, Alison G. Power, Samraat Pawar

**Affiliations:** 1 Department of Life Sciences, Imperial College London, Ascot, United Kingdom,; 2 Department of Statistics, Virginia Polytechnic Institute and State University, Blacksburg, VA, United States,; 3 Department of Biology, Stanford University, Stanford, CA, United States,; 4 Department of Biological Sciences, Virginia Polytechnic Institute and State University, Blacksburg, VA, United States,; 5 BresMed America Inc, Las Vegas, NV, United States,; 6 The Cary Institute of Ecosystem Studies, Millbrook, NY, United States,; 7 Harvard T.H. Chan School of Public Health, Boston, MA, United States,; 8 Center for Infectious Disease Dynamics and Department of Biology, Pennsylvania State University, University Park, PA, United States,; 9 Department of Integrative Biology, University of California, Berkeley, Berkeley, CA, United States,; 10 Department of Entomology, Pennsylvania State University, University Park, PA, United States,; 11 Department of Ecology and Evolutionary Biology, Cornell University, Ithaca, NY, United States

**Keywords:** vector-borne disease modeling, traits, population dynamics, transmission, vector ecology, reproductive number

## Abstract

Many important endemic and emerging diseases are transmitted by vectors that are biting arthropods. The functional traits of vectors can affect pathogen transmission rates directly and also through their effect on vector population dynamics. Increasing empirical evidence shows that vector traits vary significantly across individuals, populations, and environmental conditions, and at time scales relevant to disease transmission dynamics. Here, we review empirical evidence for variation in vector traits and how this trait variation is currently incorporated into mathematical models of vector-borne disease transmission. We argue that mechanistically incorporating trait variation into these models, by explicitly capturing its effects on vector fitness and abundance, can improve the reliability of their predictions in a changing world. We provide a conceptual framework for incorporating trait variation into vector-borne disease transmission models, and highlight key empirical and theoretical challenges. This framework provides a means to conceptualize how traits can be incorporated in vector borne disease systems, and identifies key areas in which trait variation can be explored. Determining when and to what extent it is important to incorporate trait variation into vector borne disease models remains an important, outstanding question.

## INTRODUCTION

Vector-borne diseases (VBDs) remain a serious threat to human health (San Martín et al., 2010; Dick et al., 2012; Lee et al., 2013; Mead, 2015; CDC, 2016; Faria et al., 2016), livestock (Wilson and Mellor, 2009), and agriculture (Taylor et al., 2016). Cycles or episodes of VBD disease incidence are driven by a system of interconnected vector, host, and pathogen population abundances that vary over time and space. Evidence indicates that the behavior and life history of the vector is a key determinant of any VBD’s dynamics because it influences pathogen transmission rates between vector and host individuals. These aspects of vector biology can be described as functional traits (hereafter, “traits”): measurable features of an individual organism that determine its fitness (lifetime reproductive output) (McGill et al., 2006; Gibert et al., 2015). As a result, variation in these traits between individuals, and within individuals over time determines the abundance (a measure of population-level fitness) of the vector population.

Ecological studies show that trait variation is ubiquitous and alters population, community and ecosystem level processes, accentuated by underlying non-linearities in the way individuals interact with conspecifics, other species, and the environment (Norberg et al., 2001; Imura et al., 2003; McGill et al., 2006; Agashe, 2009; Gibert et al., 2015). For example, intraspecific variation in foraging traits of single consumer species can change abundance dynamics of prey across multiple trophic levels in food webs, with the effect often being comparable to, and sometimes stronger than, adding new consumer species (Des Roches et al., 2018). Similarly, because vector-vector, host-vector and vector-pathogen interactions are non-linear, even small within-population and over-time variation in vector traits can have significant impacts on disease dynamics due to compounding effects (Lloyd-Smith et al., 2005; Martin et al., 2019). Furthermore, the traits of ectotherms vary directly and non-linearly with fluctuations in environmental conditions. This impact of trait variation on VBD dynamics is important because the resultant vector population dynamics typically occur at time scales comparable to pathogen transmission dynamics (May and Anderson, 1979; Downs et al., 2019). Finally, both vector abundance and transmission dynamics occur at faster timescales than at which hosts operate because development and generation times scale negatively with body size, and vectors are orders of magnitude smaller than their (plant or animal) hosts (Gillooly et al., 2002; Savage et al., 2004). For this reason, vector trait variation may potentially be more important than variation in host traits.

Variation in vector traits can change VBD dynamics not just by changing vector abundances but also directly by affecting the transmission rate. The full suite of vector traits can be classified into three categories in the context of VBDs. First, traits such as vector competence (ability to transmit the pathogen to host) and susceptibility directly impact disease dynamics by altering the rates at which the pathogen is transmitted and vectors and hosts become infected. Second, life history traits such as individual fecundity and longevity determine the number of susceptible vectors that enter the system. Third, interaction traits such as biting and body velocity affect transmission dynamics both directly by determining vector contact rates with hosts, and indirectly through the impact of interactions with other species on vector population dynamics. While it is accepted that vector traits are important for transmission and are temporally and spatially variable (Smith et al., 2014), for tractability, most empirical and theoretical VBD studies include only a small subset of the full range vector traits—most commonly, adult vector biting rate, mortality rate and competence (Rabinovich and Himschoot, 1990; Caraco et al., 2002; Jeger et al., 2004; Smith et al., 2012; Reiner et al., 2013; Shimozako et al., 2017; Van den Bosch and Jeger, 2017).

The need to predict disease dynamics over long timescales is critical given the rapidly changing world we live in. Statistical models (e.g., based on time series analyses) can forecast disease dynamics on the short term based on historical and contemporary dynamics. For example, the number of dengue cases in a single transmission season can be explained using statistical models that do not include biological or environmental information (Johansson et al., 2019). However, these methods are phenomenological, and make unreliable predictions over longer timescales when disease dynamics are driven by underlying non-linearities compounded by trait variation and changing environmental conditions. In contrast, mechanistic models, which capture underlying processes can improve our ability to predict VBD dynamics at longer temporal and larger spatial scales, as is the case more generally for the dynamics of ecological systems (Getz et al., 2018). Arguably, mechanistic models of VBD dynamics that capture temporal and spatial changes in vector trait variation have even greater potential to predict disease dynamics further into the future.

However, mechanistic, trait-based VBD research faces two major challenges. First, for most vector species, we lack data on how traits underlying transmission model parameters vary, forcing models to use inaccurate parameter values (for example, using the time it takes a mosquito to produce a clutch of eggs to infer biting rate) or use data from related species to parameterize models (for example, Mordecai et al., 2013; Johnson et al., 2015). Second, while trait variation is increasingly being incorporated in various ways into VBD models, we require a conceptual framework to prioritize ways in which this complex problem can be tackled both empirically and theoretically. Here, we present such a conceptual framework, with hope that it will help the research community better tackle the challenge of developing a trait-based VBD approach by summarizing the types of trait data needed for model development, and providing a general modeling scaffold that can be adapted for many focal VBD systems and questions. Many existing VBD models represent simplifications or special cases of this general framework.

This is the ideal time to overcome the challenge of developing trait-based VBD research. Recent public health crises have spurred government agencies to support the collection of large amounts of data on VBD systems, including vector traits. This, combined with innovations in empirical data collection and sharing, means that the necessary data for parameterizing and validating trait-based models are now becoming available. At the same time, the field of trait-based research is rapidly developing across the broader field of ecology, with both the theory and experimental methods growing apace (McGill et al., 2006; Pawar et al., 2015b). The many areas of ecology that are currently striving to mechanistically incorporate trait variation to understand emergent community or ecosystem level dynamics and functioning (e.g., Díaz et al., 2007; Blanchard et al., 2012; Pawar et al., 2015a; Kissling et al., 2018) provide empirical and theoretical methods that could be leveraged for VBD research.

In what follows, we non-exhaustively review the empirical evidence for trait variation and covariation and previous efforts to incorporate these types of trait variation into VBD dynamics. We highlight gaps in current trait-based approaches, including the types of trait variation and covariation that have been overlooked. We then illustrate how a mechanistic vector trait-based approach can provide new insights into VBD dynamics, and present a conceptual framework that unites most previous approaches and fills existing gaps. We end with a discussion of key empirical and theoretical challenges in the way of operationalizing trait-based VBD dynamics approaches.

## VARIATION IN TRAITS OF DISEASE VECTORS

**FIGURE 1 |:**
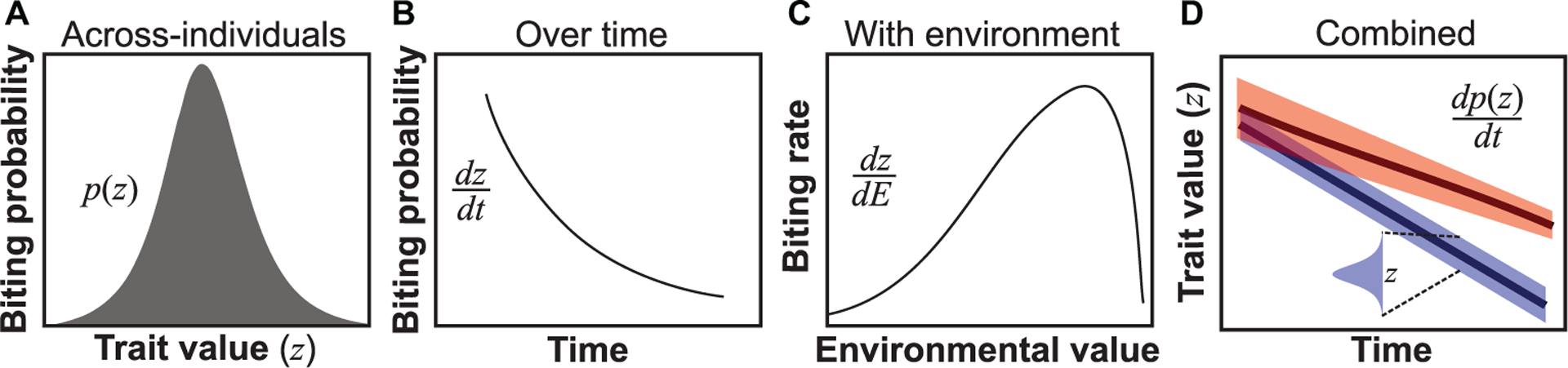
Types of trait variation found in all VBD systems. (**A**) Across-individuals: variation in a trait (*z*) within a population within a temporal snapshot, here illustrated using the probability of biting of individuals in a population at a particular age (Cator et al., 2013). **(B)** Individuals over time: for example, biting probability may vary over an individual vector’s lifespan (Cator et al., 2013). Such variation can be represented as a continuous time-dependent function *dz*/*dt*, where *dz* is the differential change in trait variation change with time (*dt*) of an individual. **(C)** Environmentally driven: For example, biting rate varies unimodally with temperature (Dell et al., 2011; Mordecai et al., 2013). Such variation is quantifiable as a continuous environmental state-dependent function, *d*z/*dE*. **(D)** Combined variation: The three types of trait variation may appear in combination. For example, across individuals in the population, trait variation may change over time both in terms of trait mean and variance (upper line) or just the mean (lower line). Although we use derivatives to represent over-time, with-environment and combined types of trait variation, in reality, it may not always be possible to express these as smooth functions for empirical reasons.

Each vector trait may vary in three primary dimensions: across individuals in a population at a time-point or interval; over time within an individual; and in response to environmental conditions (Figure 1). Note that throughout this paper, we often use both the terms “trait” and “parameter” for the same property of a vector. This is because when used in a VBD model directly, a trait is also a parameter. Thus, mortality rate and fecundity are parameters in VBD models, but are also traits because they are directly measurable. In contrast, vectorial capacity, for example, is not a trait as it is a derived measure and cannot be directly measured.

### Across-Individual Variation

The traits of individuals in a population typically vary within any temporal snapshot, either because of genetic variation, phenotypic plasticity, or both (Agashe, 2009; Bolnick et al., 2011). In general, heterogeneity in individual transmission potential can have large consequences for disease dynamics (Woolhouse et al., 1997; Lloyd-Smith et al., 2005). In VBD systems in particular, variation across individual vectors in traits such as biting rate, host preference, and longevity can lead to subgroups of the vector population having disproportionate effects on mean population fitness, abundance, transmission potential, and ultimately disease dynamics. Evidence for this kind of trait variation in vectors includes: variation among individuals in the extrinsic incubation period (EIP; time required to become infectious) (Ohm et al., 2018); nutritional status-driven variation in vector competence and behaviors linked to transmission (Takken et al., 2013; Shapiro et al., 2016); and body size-driven variation in feeding, assimilation, and respiration, and therefore development, mortality, and transmission rates (Renshaw et al., 1994; De Xue et al., 1995; Kindlmann and Dixon, 2003) (as expected from Metabolic Theory of Ecology; Brown et al., 2004; Savage, 2004; Amarasekare and Savage, 2012). Individual variation in age-specific mortality is particularly important for transmission (Clements and Paterson, 1981; Harrington et al., 2001, 2008; Styer et al., 2007). One such source of variation is infection status itself, which can generate a distribution of traits within a population. For example, recent evidence from several different systems has demonstrated that infected vectors exhibit altered foraging behaviors (Murdock et al., 2017; Eigenbrode et al., 2018). In such cases, assuming average values for traits such as biting rate can lead to significant underestimations of transmission potential (Cator et al., 2014).

### Variation Over Time in an Individual

The trait values of any given individual in a population typically also vary over time, typically due to physiological, morphological, or behavioral changes driven by ontological development or senescence. For transmission to occur, a vector must survive long enough after acquiring the parasite to become infectious (extrinsic incubation period, EIP), which can be a large proportion of the vector lifespan. Older vector individuals are: (i) more likely to be infected because they are more likely to have been exposed, (ii) more likely to be infectious because they are more likely to have survived EIP, and (iii) are more likely to transmit the pathogen onward because they are alive to bite subsequent hosts after becoming infectious. Therefore, variation in vector lifespan itself as a trait can disproportionately contribute to transmission. There is evidence for age-specific changes in vector immune function (Christensen et al., 2005; Hillyer et al., 2005; Laughton et al., 2014), flight performance (Nayar and Sauerman, 1973), feeding behavior (Alto et al., 2003; Den Otter et al., 2008; Bohbot et al., 2013), mortality rates (Bellan, 2010) and competence (Soliman et al., 1993). When multiple life stages of the vector contact hosts (e.g., ticks), transmission efficiency may also vary with stage (Caraco et al., 2002; Coletta-Filho et al., 2014). All these time-dependent changes in vector traits could lead to significant variation in the number of infectious vectors and their contact rates with hosts.

### Environmentally Driven Variation

The majority of vectors are small ectotherms, so their behavioral, life history, and interaction traits environmentally sensitive. Variation due to environmental drivers may have both short- or long-term effects on vector traits. At present, most of the data on this kind of variation come from studies on temperature as a driver. In particular, numerous studies have measured effects of variation in environmental temperature on vector life history traits (Kersting et al., 1999; Bayoh and Lindsay, 2003; Delatte et al., 2009; Ciota et al., 2014) and competence (Kramer et al., 1983; Murral et al., 1996; Dohm et al., 2002; Paweska et al., 2002; Wittmann et al., 2002). Other environmental variables such as humidity, precipitation, and nutrient availability also directly affect vector traits at different life stages (Wittmann et al., 2002; de Costa et al., 2010; Takken et al., 2013; Shapiro et al., 2016). However, compared to temperature, fewer data exist on these drivers, and models that incorporate these other variables are faced with a significant parameterization challenge. In Section 3 below, we address this issue further in the context of past modeling approaches to capture environmental effects on VBD dynamics.

### Mechanistic Covariation Between Traits

Most traits covary with others because they are mechanistically linked through physiology (Charnov, 1993; Brown et al., 2004). This is very much true for vectors as well. For example, mosquitoes infected with bird malaria parasites exhibit reduced fecundity, which in turn increases longevity (Vézilier et al., 2012). This kind of trait covariation often appears in the form of life-history trade-offs (Charnov, 1993) and affects both vector population abundance and disease transmission rate. This is important because covariation between (mechanistically linked) traits implies that variation in a trait indirectly related to disease transmission, such as fecundity, can influence horizontal (host to host) transmission of pathogens by influencing another trait that does, such as biting rate (fecundity and biting rate covary positively). Also, covariation between life-history traits such as adult lifespan and fecundity (which covary negatively) can change VBD dynamics indirectly by altering vector population dynamics. Therefore, it is important for trait-based transmission models to account for mechanistic covariation between traits. Indeed, there is recent evidence that accounting for this can yield new insights into disease dynamics. In particular, recent work using Metabolic Theory of Ecology to incorporate trait variation into micro- and macro-parasite disease transmission has resulted in models that more accurately capture disease dynamics, by linking traits connected to metabolic rate, such as fecundity and mortality rate (Molnár et al., 2017; Kirk et al., 2018, 2019). To our knowledge, no such examples currently exist in VBD research, but similar efforts there are likely to prove fruitful.

## EXISTING APPROACHES FOR INCORPORATING TRAITS INTO VECTOR-BORNE DISEASE DYNAMICS

Here we provide a brief overview of how traits and trait variation have been incorporated into mathematical models of VBD dynamics to put our conceptual framework (Section 5 below) into context and identify gaps in existing knowledge.

### Classical Compartment Models

Classical compartment models focus on the proportion of different (Susceptible, Exposed, Infected, Recovered) sub-populations of the host and vector, assuming total abundances of the two species are constant. For example, the Ross-Macdonald model for malaria transmission by a mosquito (SI Section 1), and its extensions (including for non-mosquito vectors) (Macdonald, 1957; Smith et al., 2012; Reiner et al., 2013) focuses exclusively on the parameters governing the transmission rate of the pathogen between susceptible and infected vector and host subpopulations, most of which are mosquito traits. It yields a relatively simple equation for the basic reproduction number of the disease (*R*_0_)—the number of new infectious cases that would arise from a single infectious case introduced into a fully susceptible host population—which quantifies its transmission potential or risk (Macdonald, 1957; Smith et al., 2012) (see SI section 1 for derivation):

(1) $$R_{0}={\left(\frac{Va^{2}bce^{-\mu P}}{Hd\mu }\right)}^{\frac{1}{2}}$$

Here, *V* is vector density, *a* is per-vector biting rate (bites/day), *b* is the proportion of the bites by infective mosquitoes that produce infection in susceptible humans, *c* is the proportion of bites by susceptible mosquitoes on infectious humans that infect mosquitoes (thus, *bc* is vector competence), *μ* is adult vector mortality rate, *P* is the extrinsic incubation period (pathogen incubation period within the vector), *H* is host density, and *d* is the rate at which infected hosts recover and acquire immunity. Note that equation 1 emerges from an extension of the original Ross-Macdonald model, which did not include vector competence or EIP of the pathogen (*P*) in this way.

Thus, classical compartment models incorporate some vector infection and life history traits; in the above example, biting rate, vector competence, extrinsic incubation period, and mortality rate. However, these traits are assumed to be independent of each other despite the fact that they covary (e.g., mortality and biting rates), with potentially compounding effects on transmission. Further, compartment models generally assume that vector and host traits do not affect total vector population size, and that these traits do not vary across individuals, over time, or across environments (Figure 1). We note that there is some debate about the precise form of the *R*_0_ equation based on classical compartment models because its exact form depends on the method used to derive it (Li and Blakeley, 2011). We used the next-generation matrix method (SI section 1). However, all versions of *R*_0_ are just different convolutions of the same parameters or traits as in equation 1, and all assume that traits to not vary or covary.

Classical compartment models have been extended to incorporate vector population dynamics by adding vector life-stage compartments (Anderson and May, 1979; May and Anderson, 1979; Hoshi et al., 2014; Johnson et al., 2018; Ng et al., 2018) (Anderson-May type models). These models introduce additional parameters or traits for vector life history, which correspond to directly measurable vector traits such as mortality and fecundity, or parameters that can be derived from stage-specific survivorship and development time (see SI section 1.2). In these studies as well vector trait variation and co-variation were not initially considered.

### Extensions of Classical Compartment Models That Include Trait Variation

We now consider extensions of classical compartment models that have included trait variation. These can be classified into a few distinct categories that have tacked different aspects of the challenge of a fully trait-based VBD study.

### Classical Compartment Models With Trait Variation

Several studies have incorporated trait variation directly into the classical compartment (Ross-Macdonald type) models. For example, extensions of the Ross-Macdonald model to incorporate parasite latency in malaria vectors are common (Reiner et al., 2013). Such efforts have led to several new insights. Specifically, several studies have shown that variation in single traits such as age-specific vector mortality drives changes in the predicted sensitivity of *R*_0_ to vector control (Styer et al., 2007; Bellan, 2010; Novoseltsev et al., 2012). There are also many studies showing how variation in single life history traits, such as longevity or biting rate, associated with infection (McElhany et al., 1995; Koella, 2005; Lefèvre and Thomas, 2008) or nutrition (Shapiro et al., 2016) affects transmission. More recently, it has been reported that incorporating individual variation in EIP in mosquitoes derived from empirical data leads to elevated risk of dengue (Kamiya et al., 2019). Nevertheless, across all these studies, variation in certain traits, such as heterogeneity in host-vector contact rate or vector traits such as fecundity tend to be systematically overlooked (Reiner et al., 2013).

### Anderson-May Type Models With Trait Variation

In another class of studies, aspects of vector ecology have been added to Anderson-May type models (classic compartment models combined with vector life stage compartments) (SI section 1.3). These aspects include environmental drivers (Beck-Johnson et al., 2013; El Moustaid and Johnson, 2019) and species interactions (Depinay et al., 2004; Nakazawa et al., 2012). Crowder et al. (2019) modeled the effect of species interactions on transmission of persistent and non-persistent plant pathogens by assessing the predicted impact of mutualistic, predator-prey, and competitive pressures on vector fecundity, mortality, and movement. They found that species interactions can alter the rates of pathogen spread in these systems through changes in vector movement (Crowder et al., 2019). This is one of few examples where there has been an effort to include the third class of vector traits—species interaction traits. In our proposed framework below, we illustrate how this class of interaction traits can be incorporated into modeling and empirical studies, and emphasize the potential importance of doing so. Most recently, environment-driven trait variation has been incorporated by modeling the effects of precipitation and temperature on multiple vector traits. In these studies, traits are allowed to covary across environmental states because they share a common driver, but nevertheless, are not explicitly, mechanistically linked (e.g., in the form a tradeo between adult fecundity and survivorship). For example, Parham and Michael (2010) derived an equation for vector population size as a function of traits using a statistical approach, then allowed these traits to vary as functions of environmental conditions. Mordecai et al. (2013,^2017^,^2019^) built upon this approach to include empirically derived, non-monotonic thermal responses for life-history and transmission traits. Brand et al. (2016) took a similar approach by allowing biting rate and EIP parameters to depend on temperature. Other studies have used empirically derived relationships of density-dependence of individual vector traits (e.g., mortality) (Caraco et al., 2002; Hancock et al., 2016; Caminade et al., 2017; Siraj et al., 2017; Liu-Helmersson et al., 2019).

### Classic Compartment and Anderson-May Type Models With Individual-Level Trait Variation

Another class of studies has simultaneously incorporated individual-level variation in multiple vector transmission and life-history traits into classic compartment or Anderson-May type VBD models. Some of these studies also include the time axis of individual trait variation (Figure 1B). For example, Brand et al. (2016) determined the number of infectious bites delivered by midges by combining the EIP of bluetongue virus, age-specific biting rate, and mortality. They found that calculating model parameters from trait variation in this way can dramatically change *R*_0_ and the estimated impact of vector control. However, while midge age-specific biting rate and survival were used to determine whether an individual survives through EIP, the model does not mechanistically link these two traits. Similarly, Brady et al. (2016) incorporated variation in adult female mosquito blood feeding, egg laying, and accounted for differences in larval ecology (but not explicitly as larval traits) to re-calculate vectorial capacity, and showed that this increased the relative importance of larval vector control methods. Thus, in all these efforts, fine-scale, often individual-level variation in traits has been incorporated, but the traits are still not mechanistically linked, which we argue is fundamentally important to emergent VBD dynamics. This shortcoming has been addressed to a degree by studies that use individual or agent-based models to simulate trait variation across individuals, allowing population level properties to emerge “naturally” and drive VBD dynamics. This individual-based approach implicitly includes trait covariation, and in a variety of VBD systems has provided key insights into the role of trait variation in these systems (Rabinovich and Himschoot, 1990; Focks et al., 1993a,b; Bomblies et al., 2008; Eckho, 2011; North et al., 2013; Killeen and Chitnis, 2014). However, these studies rely on computer simulations that are not analytically tractable, and require very detailed biological data for accurate parameterization. For example, there is no straightforward way to determine which traits are important by performing an elasticity or sensitivity analysis (e.g., Brady et al., 2016; Section 4.1 below). They are useful in that they provide predictions tightly linked with biological data in specific scenarios or systems, but do not yield generalizable information about the relationships between parameters and transmission.

In summary, vector trait variation has been incorporated in various ways in a large body of previous studies. This has provided important insights into how much vector population dynamics matter to VBD dynamics. However, most studies do not address trait variation and mechanistic links between traits systematically or comprehensively, and typically exclude a potentially important class of trait variation in the form of interaction traits. Below, we present a framework for incorporating the effects of the full suite of vector trait variation and covariation, through the vector’s individual fitness and abundance dynamics, to VBD dynamics. Our objective is not to encourage any one study to tackle the full scope of the challenge inherent in a fully trait-based approach, but to provide a conceptually unified framework that puts into context previous efforts, and which can help future theoretical and empirical studies to prioritize which aspects of the challenge to tackle first. It can also provide a general modeling scaffold that can be adapted for focal VBD systems and questions. Ultimately, we hope that this will facilitate the development of approaches for modeling and empirically validating fully trait-based VBD systems. To motivate the need for trait-based approaches, we first provide an example to illustrate how mechanistically incorporating traits into VBD models can lead to novel predictions about vector population dynamics and therefore transmission.

### INCORPORATING TRAIT VARIATION MECHANISTICALLY INTO VBD MODELS: AN EXAMPLE

**FIGURE 2 |:**
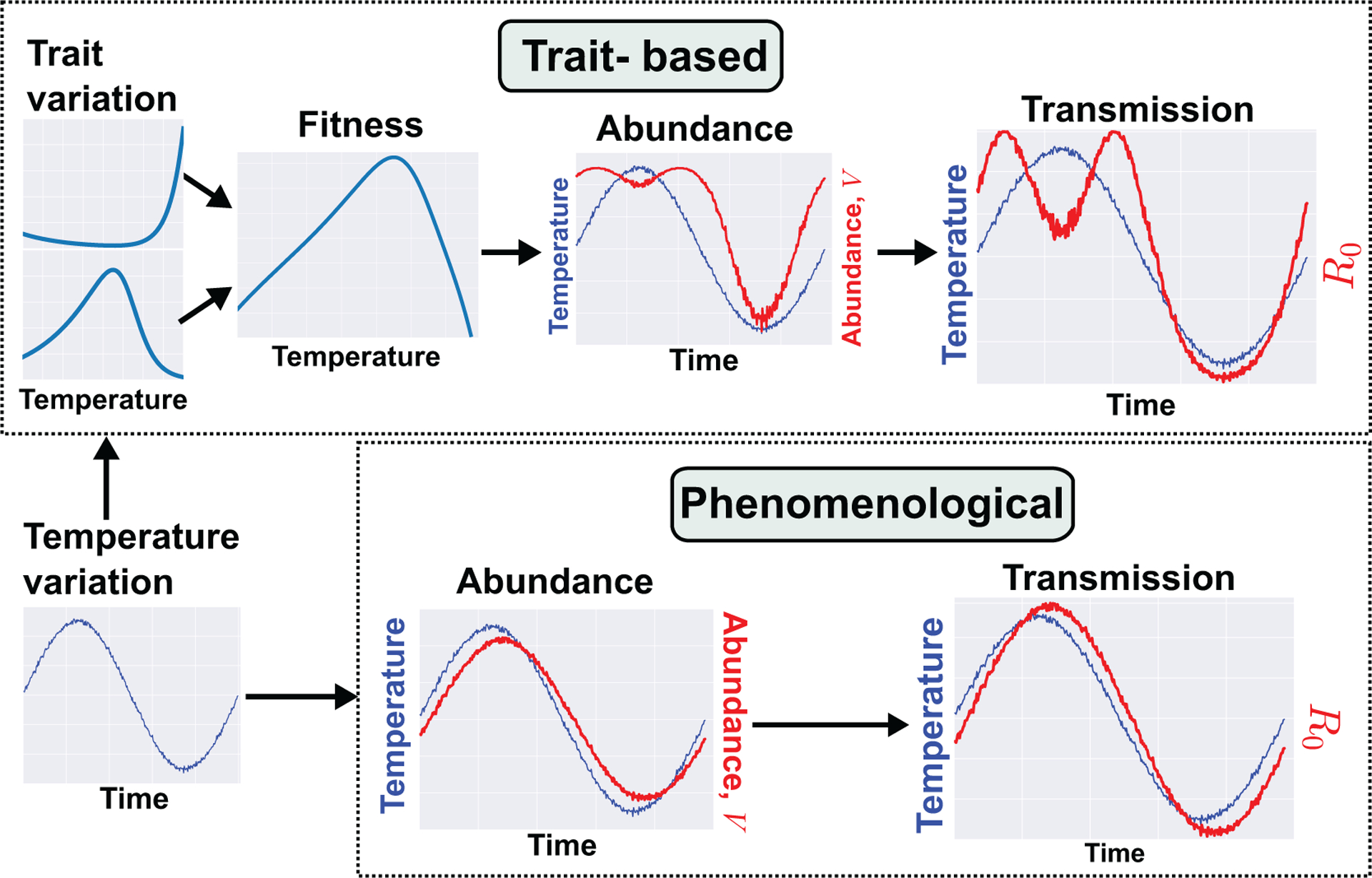
An example trait-based model for malaria transmission. We illustrate here the contrast in models and resulting dynamics produced from trait-based vs. phenomenological approaches. Both models cover a time scale of one year and seek to predict the fluctuation in transmission risk or rate (*R*_0_) during that period. Full details of both models can be found in SI section 3.

We use the effect of temperature on trait variation (a type of environment-driven variation; Figure 1C) and model its effect on transmission as an example, and show how a mechanistic trait-based approach can be used to understand the importance of specific traits though sensitivity analyses. Temperature is a ubiquitous driver of trait variation in both adult and juvenile vector traits. To incorporate this trait variation and co-variation among traits into transmission, we model the effects of temperature-driven life-stage specific trait variation on vector population dynamics by deriving a mechanistic model for population density, *V*. This model applies to any class of vector with holometabolous life stages such as mosquitoes. We consider this trait-based abundance model to be mechanistic because, for example, unlike the statistical model derived by Parham and Michael (2010), it depends explicitly on the vector’s intrinsic growth rate, *r*_m_, which is itself derived from multiple traits using life-history theory. Full details of the model are provided in SI section 3. Briefly, *r*_m_ is a function of adult peak fecundity (*b*_pk_), age-related fecundity-decline rate (*κ*), adult mortality rate (*μ*) and juvenile development time (*α*) and juvenile mortality (*μ*_J_). Variation (and co-variation) in each of these traits across temperature is characterized by the thermal performance curve of each trait. By incorporating such environment-driven trait variation into a vector population abundance model we can derive the *R*_0_ of the disease dynamic over time (Figure 2). The transmission compartments of the model can apply to a wide range of pathogens and parasites.

We contrast this trait-based model with a phenomenological modeling approach that has been used in previous studies. Under the previous approach, abundance (*V*) is directly associated with temperature by fitting a time-series model or where abundance is assumed, *a priori*, to follow a sinusoidal function (Bacaër and Guernaoui, 2006; Bacaër, 2007; Bacaër and Ouifki, 2007; Bacaër and Ait Dads, 2012; Hoshi et al., 2014; Johnson et al., 2018; Ng et al., 2018). This results in a disease dynamic where *R*_0_ tracks temperature variation with some time lag (Figure 2). In contrast, using the trait-based approach that maps traits through parameters to vector population size, vector populations emerge earlier in the year and persist later into the cooler late summer season with a dip in the warmest period of the summer. These differences in *V* in turn extend the period of annual transmission with an early and late summer peak. The trait-based model predicts a longer transmission season than the phenomenological model and a decrease in transmission risk in the warmest period. The latter result contradicts the general “warmer is better for vectors” view (also see Mordecai et al., 2013). These results are also consistent with those of Molnár (2013), who used metabolic theory to mechanistically model the effect of temperature-driven trait variation on infection rate of an endothermic host by a nematode parasite. They found that a continuous spring-to-fall transmission season morphed into two distinct transmission seasons as the climate warmed. In both cases, these novel predictions arise from mechanistically linked trait thermal performance curves, in contrast to the simpler sinusoidal seasonal forcing of vector population size. The similarity in predicted disease dynamics across these very different systems suggests that mechanistically incorporating trait variation can reveal general constraints on VBD systems — in this case, the effect of temperature on VBD dynamics through life-history traits.

The example we have developed here also illustrates a key theoretical point we raised at the start. If vector traits change at the same or shorter timescales (here, driven by within-year temperature change) than the rate of pathogen transmission, the classical approach will fail to capture important aspects of contemporary and future disease dynamics (Anderson and May, 1981; Heesterbeek and Roberts, 1995; Bacaër, 2007) because they do not capture how variation in key vector traits or parameters (e.g., *a, b, c, μ*) interact, and how this sets the timescales of the dynamics. In our example, this timescale of abundance fluctuations, set by the inverse of growth rate *r*_m_ arises from the mechanisms built in via the underlying traits.

### A Trait Sensitivity Analyses

**FIGURE 3 |:**
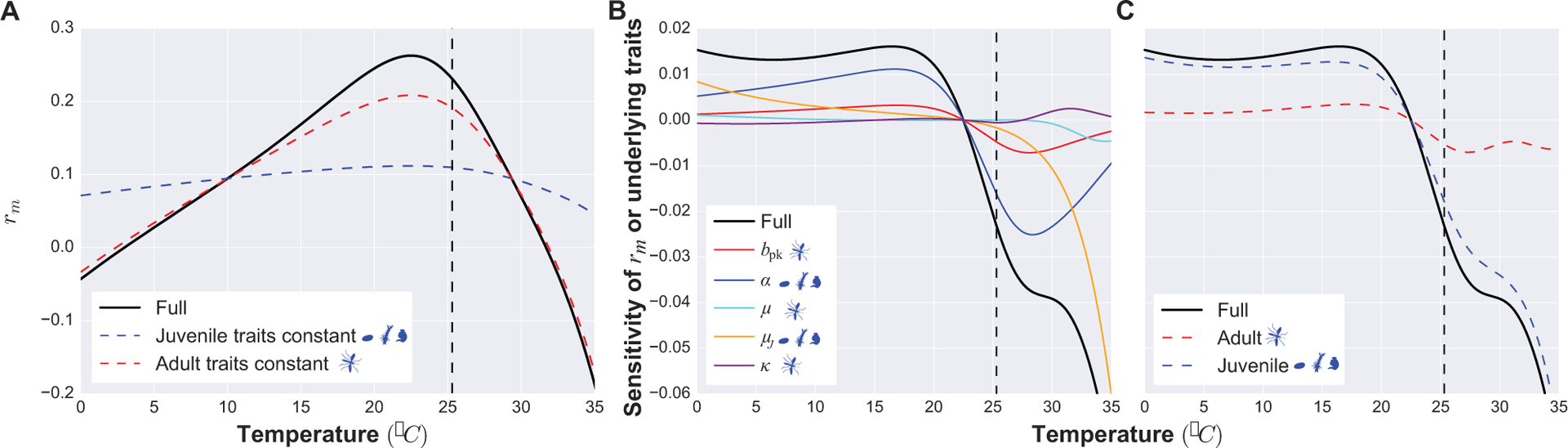
An example vector trait sensitivity analysis. **(A)** The temperature-dependence of maximal growth rate (*r*_m_) (black line) and its sensitivity to adult (peak fecundity *b*_pk_, age-related fecundity decline rate *κ* adult mortality rate *μ*; red dashed line) and juvenile traits (juvenile development time *α* and mortality *μ*_J_; blue dashed line) combined. When juvenile traits are held constant with respect to temperature, the temperature dependence of *r*_m_ changes more substantially (deviation of the blue dashed curve from the black one) than when adult traits are held constant (deviation of the red dashed curve). Thus juvenile traits have a stronger influence than adult traits in shaping the response of population fitness to temperature. The vertical dashed line marks the thermal optimum of fitness. To the left of this temperature the fitness of the population is increasing (when *r*_m_ *>* 1 the population is growing). To the right of the thermal optimum the fitness of the population starts to decrease (when *r*_m_ *<* 1 the population is declining). **(B)** The sensitivity of *r*_m_’s temperature dependence to that of each underlying trait can be assessed by decomposing the derivative of *r*_m_ with respect to temperature (black line) into partial contributions (the differently colored lines) of each trait’s temperature dependence (using the relationship $\frac{dr_{m}}{dT}=\;\frac{\partial r_{m}}{\partial b_{pk}}\frac{\partial b_{pk}}{\partial T}+\frac{\partial r_{m}}{d\alpha }\frac{d\alpha }{dT}+\frac{\partial r_{m}}{d\mu_{}}\frac{d\mu }{dT}+\frac{\partial r_{m}}{d\mu_{\text{J}}}\frac{d\mu_{\text{J}}}{dT}+\frac{\partial r}{dk}\frac{dk}{dT}$. Here the curves of the traits closest to the black curve contribute more to the temperature sensitivity of *r*_m_ (thus, development rate and juvenile mortality have the strongest contributions). When a curve has a positive value on the y-axis (positive derivative), it means that the trait increases with temperature in that temperature range (as can be seen in A; also see SI section 4.1). Temperatures where the curve is negative are temperatures at which the trait value is decreasing as temperature increases. **(C)** The same result as B, but traits combined by life stage, as in A. Full details of the trait sensitivity analysis are in SI Section 4.

A major advantage of a mechanistic trait-based approach is that it allows investigation of the impact of different, covarying traits on disease dynamics through underlying (fitness) effects on population growth and abundance. For example, a trait sensitivity analysis of the population intrinsic growth rate, *r*_m_, in the above trait-based model allows us to investigate the relative importance of juvenile versus adult traits in determining effects of temperature on abundance (and therefore transmission) (Figure 3, SI section 4). This leads to a key insight: juvenile traits are expected to play a major role in determining vector intrinsic growth rate, abundance, and transmission across temperatures. In particular, the thermal sensitivity of abundance (*V*) and the underlying population intrinsic growth rate (*r*_m_) is driven by the temperature-driven variation in larval stage traits. Such results provide quantitative targets for validation using field data. Sensitivity analyses of transmission measures with respect to traits also allow key traits to be identified, guiding further empirical and theoretical work on the contributions of traits to VBD system dynamics. For example, hypotheses can be tested about how different control strategies targeting specific traits could work using such a model.

## TOWARDS A TRAIT-BASED FRAMEWORK FOR VBD RESEARCH

**FIGURE 4 |:**
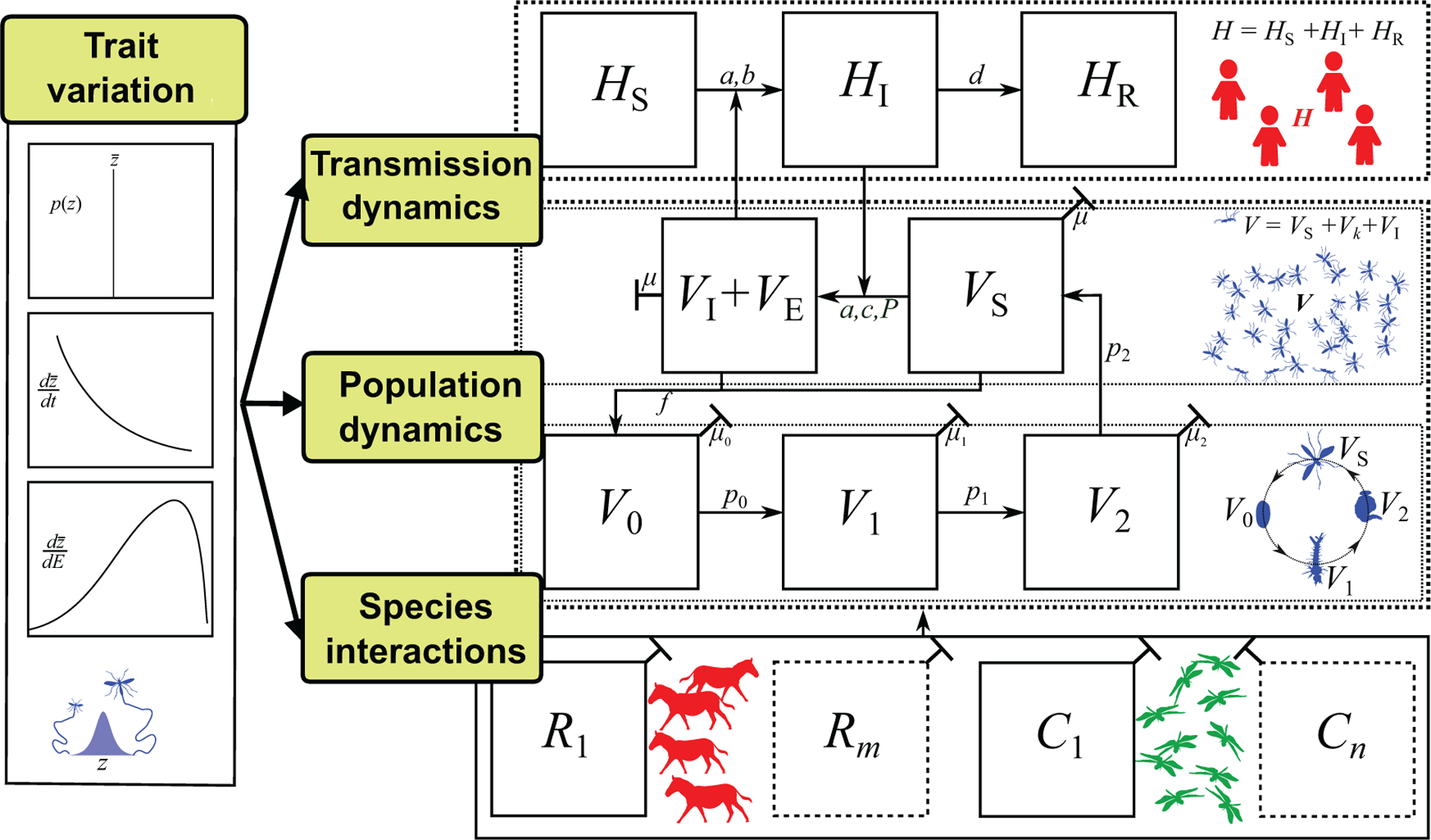
The trait-based framework for vector-borne disease systems. For illustration, we have used mosquitoes as the vector, but this framework can be applied to any vector with distinct stage or age classes (further details in SI). Arrows between panels represent parameters (potentially with underlying traits) that determine population or disease dynamics. Disease dynamics compartments: Number of Susceptible (*S*), Infected (*I*), and Recovered (*R*) hosts (*H*_S_, *H*_I_, *H*_R_, respectively; additional compartments, *H*_J_, can be added) and number of Susceptible, Infected, and Exposed (E) vectors (*V*_S_, *V*_I_, *V*_E_ respectively). Vector population dynamics compartments: number of vector individuals at egg, larval pupal and adult stages (*V*_0_, *V*_1_, *V*_2_, *V*_S_ respectively; additional compartments *V*_k_ can be added); Species interaction compartments: Abundance of a single resource species (*R*_1_) that is the primary energy source of the vector population (may actually be the host itself, i.e., *R*_1_ = *H*_*S*_), and a single consumer species (*C*_1_) that is the primary source of mortality for the vector population (additional species can be added); Trait variation: A suite of trait to parameter mappings that determine vector population fitness (e.g., vector mortality, fecundity and biting rates), and a single type of trait variation, such as variation with an environmental factor (*dz*/*dE*; e.g., temperature; see Figure 3). For developing a mathematical model of such a system, the most common tool would be ordinary differential equations (ODEs), as illustrated in SI Sections 1–2.

As the above example illustrates, incorporating trait variation mechanistically in VBD models can capture novel dynamics and provides the opportunity to investigate the relative importance of individual traits for VBD transmission. In the field of VBD dynamics research, a framework for implementing such a trait-based approach is largely missing. We now present a framework for incorporate infection, life history, and interaction traits— and variation in these traits within individuals, populations, and across environments—into models of VBD dynamics. This scaffold can be adapted for any focal VBD system, trait(s), and environment of interest to ask specific questions about how trait variation affects dynamics. The framework is illustrated in Figure 4, with a more detailed description in SI Section 2.

In general, a fully trait-based VBD model or empirical study should contain all of the following elements, but with the level of detail and model complexity depending on the system and research questions of interest:

1. **Transmission compartments for each focal host and vector species:** For example, the SIR compartments as in the Ross-Macdonald type models (e.g., *H*_S_, *H*_R_) with additional *j* host sub-compartments (*H*_J_) specified for particular systems.
2. **Vector life history compartments:** These would include the commonly used susceptible and infected (adult) vector sub populations (*V*_S,_ *V*_I_), as well as vector juvenile life stage subpopulations, starting at birth (*V*_0_) and followed by *l* immature stage compartments (*V*_*l*_) specified depending on the vector species. In adult stages, we include the potential for *k* additional stages (*V*_*k*_) leading to infectious adults (*V*_I_).
3. **Species interaction compartments:** These represent the abundance of species other than the vector or host that influence the VBD dynamic. This necessarily adds considerable complexity to the VBD model, but a feasible starting point would be to identify single consumer and resource populations (*C*_1_, *R*_1_) that have the biggest impact, either indirectly by modulating vector life history (e.g., mortality) or transmission (biting rate) traits, or directly by changing vector abundance. This could be altered to include other types of interactions, including competition and mutualism.
4. **Trait Variation:** A suite of traits needed to model trait to parameter mappings need to be identified as well as models for variation in those traits along at least one dimension (such as with-environment *dz*/*dE*) (Figure 1). The extent to which these traits affect vector population abundance and transmission rate can be determined with iterative model development and trait sensitivity analyses (e.g., Figure 3).
5. **Mechanistic Links Between Traits:** The traits should be mechanistically linked such that they covary in a biologically meaningful way. This may be accomplished either by developing empirically determined, phenomenological models of trait covariation, or by modeling how multiple underlying traits together affect a VBD dynamics parameter through shared bio-mechanical and metabolic constraints. We note that such covariation may not always be important, which can again be understood by iterative model development and trait sensitivity analyses.

We do not show explicit linkages between trait variation, consumer-resource, and life history sub-compartments in Figure 4 because these will vary with VBD system. For example, in the case of most aphid-transmitted diseases, the resource (*R*) and host (*H*) are often the same. For other vectors, such as *Anopheles* mosquitoes and *Ixodes* ticks, the transmission relevant hosts (*H*) may make up only a proportion of the resources (*R*) that regulate growth and reproduction (e.g., LoGiudice et al., 2003; Donnelly et al., 2015). While not all compartments presented in Figure 4 are necessary for all VBD systems or questions, the full framework provides a means to conceptualize how traits can be incorporated into specific systems and scenarios and identify which types of trait variation need to be investigated.

### Implementing Trait-Based Approaches

In practice, a trait-based framework can be broken down into four (sequential) components, each a mapping (→) to be quantified through empirical studies coupled with mathematical modeling:

1. Trait→Parameter
2. Trait-Variation→Fitness
3. Fitness→Population Dynamics
4. Population Dynamics→Disease Dynamics

We now explain each of these components and consider approaches for tackling them. We emphasize that we are not advocating that every study tackle each of these components. Specific studies or research programs may focus on all or a portion of these components depending on question being addressed and theoretical or data limitations. For example, in Section 4, we tackled steps 2–4 without considering species interactions. Additionally, tackling component 1 would have entailed deriving the life history traits *b*_pk_, *μ*, etc. and transmission traits *a*, *b*, etc. explicitly from underlying traits, instead of assuming they have particular empirically derived forms as we did (SI Section 4). We did not attempt to map traits on to parameters or incorporate species interactions because more data are needed on the mechanistic basis of parameters. This lack of data is a major challenge for trait-based approaches which we will discuss below.

### Trait→Parameter

A key component of any trait-based framework is the mapping of trait values onto mathematical VBD model parameters (Figure 4). Deconstruction of parameters into their underlying traits bounds the parameter’s feasible range (parameter space) and reveals how different parameters are linked (e.g., two parameters may share an underlying trait) and therefore may covary. Advances in biomechanical and metabolic approaches offer an opportunity to link physical and performance traits (e.g., size-scaling) and naturally link traits together mechanistically (e.g., using metabolic rates; Charnov, 1993; Brown et al., 2004; McGill et al., 2006; Amarasekare and Savage, 2012; Pawar et al., 2015b). For example, body size drives not just adult vector biting rate, but also its fecundity and mortality rates. Recent advances in metabolic modeling also offer an opportunity to determine encounter rate parameters between vectors and hosts (Dell et al., 2011, 2014; Pawar et al., 2012, 2015a; Gilbert et al., 2014; Rizzuto et al., 2018) and even capture within-host parasite dynamics (Kirk et al., 2018). To illustrate the potential of such approaches and the fundamental importance of Trait→Parameter mappings, we derive vector biting rate mechanistically using a combination of biomechanics and ecological metabolic theory (SI Section 2.1). This allows us to deconstruct biting rate into component traits, yielding new insights into how biting rate may vary with adult vector body size at emergence, and how it may co-vary with other traits such as fecundity and mortality rate. Empirical studies on specific vectors are crucial for validating such trait-parameter models. Ideally, such studies should measure multiple traits simultaneously (for example biting rate, fecundity, development time, and mortality rate) so that covariances between traits can also be validated.

### Trait-Variation→Fitness

The second key component is to use Trait→Parameter mappings to quantify how variation in a vector’s traits affects its population-level fitness: the weighted average of fitness values across its trait variants. For example, variation in and covariation between in biting rate, fecundity, and mortality would together affect population fitness (e.g., *r*_m_). Mapping any of the three types of trait variation (Figure 1) onto vector population fitness requires the re-definition of the parameters as functions (e.g., *p*(*z*), *dz*/*dt*, *dz*/*dE*; Figure 4), ideally constructed mechanistically using Trait-Parameter mappings (previous component). Our example (SI Section 3) serves to illustrate this, as we explicitly derived population fitness using environment-driven trait variation. Here again empirical studies on specific vectors that measure multiple traits simultaneously so that they can be related to vector fitness (e.g., by mapping them to maximal growth rate, *r*_m_, as we have done in Section 3) are crucial (Ohm et al., 2016).

### Fitness→Population Dynamics

The third component is to quantify how trait variation determines vector population abundance or dynamics over time through fitness. This requires the construction of dynamic models for stage-structured vector population dynamics that incorporate trait variation (Figure 4). Initial progress can be made by empirically measuring trait variation (in contrast to deriving Trait→Parameter and Trait-Variation→Fitness mappings) and plugging it into stage-structured population dynamics models (e.g., Brand et al., 2016). In our example trait-based model (Figure 2), we took such an approach, mapping empirically validated (Sharpe-Schoolfield type) temperature-dependent trait variation onto a vector fitness and abundance model. To derive more analytically sophisticated methods, two promising approaches are trait-driver theory and integral projection models. Trait driver theory uses methods inherited from quantitative genetics to study how trait variation drives abundance dynamics (Norberg et al., 2001; Webb et al., 2010; Enquist et al., 2015), but has not yet been applied to stage-structured population growth. Integral projection models (Coulson, 2012; Rees et al., 2014; Metcalf et al., 2016) are a promising approach to tackling this challenge (Smallegange et al., 2017; Struckman et al., 2019). For example, because body-size is a key physical trait that affects multiple traits and also changes with life stage (over time), integral projection models that incorporate size-driven changes in life history traits across stages would be a promising avenue for applying these methods to vector population dynamics. After initial theoretical development in this direction, additional realism such as carryover (e.g., maternal) effects across life stages may be incorporated for specific VBD systems (Lorenz and Koella, 2011).

One important element of realism that affects stage-structured population growth that we have included as optional compartments in our general framework is the effect of species interactions (Figure 4). There is increasing interest in incorporating species interactions into VBD dynamics (Keesing et al., 2010). This is an area of ongoing investigation not just in VBD research, but in ecology in general. Species interactions impact life history traits, especially fecundity and mortality, by shifting them from the baseline, interaction-independent values (Roux et al., 2015). For example, in consumer-resource interactions, fecundity increases with availability of the vector’s resources (vector-resource or vector-host interaction), and mortality with the vector’s consumers (vector-predator interaction). Tackling this challenge will require mathematical models paired with complementary empirical studies that tractably include the impacts of species interactions on baseline life history parameters, especially fecundity and mortality. One relatively simple way to make progress in this direction is allow life history parameters (e.g., development rate, fecundity, and mortality) to be affected by species interactions, circumventing the complexity of explicitly adding consumer-resource dynamics to vector population and disease dynamics.

### Population Dynamics→Disease Dynamics

The final component is to combine trait-based vector population dynamics and transmission rates into a model of VBD disease dynamics (Figure 4). To achieve this, two theoretical issues in particular need to be addressed. First, how trait variation determines the timescale of vector population fluctuations relative to the timescale of disease dynamics needs to be modeled and empirically validated. Our example (Section 4) illustrates this issue. A trait-based approach that derives the timescales of population fluctuations mechanistically would “naturally” be able to reveal whether and when the separation of the timescales of population and disease dynamics, implicit in classical (compartment-type) VBD models, is valid. In our worked example, this separation was clearly not justified. Second, in addition to vector traits that affect vector population dynamics, models and data are needed on transmission traits (e.g., biting rate *a*, *P*, *b,* and *c* in eqn. 1). In many cases, these transmission traits will be the same as those determining fitness. For example, both, encounter rate with host and with the vector’s resources (or predators) are determined by body size through velocity and detection distance (SI Section 2.1). Indeed, the host is the primary or sole resource in many vectors (e.g., aphids) which links transmission parameters directly to the vector’s fitness though biting and feeding rate.

### KEY CHALLENGES

The above components for implementing a trait-based VBD modeling approach share four key challenges to differing degrees: *dat*a- how to prioritize experiments and report data; *parameterization*- how to link model components to empirical data; *model selection* and *validation*- how to choose and validate the most parsimonious models at each step.

### Data

Data availability is a serious constraint on model development. New data collection efforts are underway in several disease systems. To achieve the most from trait studies, data should be reported at the most disaggregated level possible, including multiple, individual-level measurements over time where possible. Beyond individual studies, consolidating datasets with individual measurements into common formats would allow for the identification of gaps and coordinated data collection efforts to specifically target the traits and conditions that are data-poor. Toward this goal, we have recently launched a hub for storing and accessing vector trait data (www.vectorbyte.org) and a platform for coordinating data collection efforts (www.vectorbite.org).

### Parameterization

Accurately quantifying trait variation in vectors at the population level is a major barrier to developing models that map trait values onto vector population and disease dynamics. In addition to identifying and incorporating empirical trait measurements from the literature to match model parameters, the variation and uncertainty in the traits must be quantified. Using approaches that allow quantification and propagation of uncertainty from traits through to population and disease dynamics, such as Bayesian inference, is critical (Clark, 2007; Johnson et al., 2015). In addition, parameter sensitivity and elasticity analyses in trait-based models (Figure 3) are needed to provide insights into the variation in and covariation between traits driving VBD dynamics, and determine which traits are particularly important. For example, trait variation in both juveniles and adults may need to be incorporated simultaneously into VBD dynamics models (Figure 3).

### Model Validation and Selection

A fundamental goal of trait-based VBD research should be to determine the conditions and systems in which vector traits drive significant variation in disease dynamics and spread (e.g., through *R*_0_). This requires validation of models, ideally with data on the spatial or temporal distribution of traits or environmental drivers as predictors. In contrast to inference or calibration of a model, validation is the process of assessing how well a parameterized model can replicate data that was not used for parameter inference or calibration (i.e., out-of-sample prediction) (Hooten and Hobbs, 2015). Validation of the entire framework will require data on trait variation and population growth rates, abundance variation and disease incidence data over space and/or time. It is not realistic for one study to accomplish this. However, the advantage of this component-based approach is that each component embodies a meaningful research direction that can stand alone.

Ultimately, much of the potential complexity across all the components of a trait-based VBD modeling approach arises from the number of trait and variation types that need to be considered for building a minimal adequate model for a given system and scenario. This reemphasizes the importance of sensitivity analyses to determine the adequate level of complexity.

Overall, model complexity is a major hurdle that trait-based approaches will need to tackle. We emphasize that previous studies have lacked the massive data on both traits and abundances that are now becoming available. There has been a recent burst in the development of methods to predict population dynamics by using traits such as metabolic rate, fecundity, mortality and inter-species interaction rates, to constrain model parameters. We are advocating for a more concerted effort to use these advances in the field of VBD research. Depending on the availability of data (e.g., on species’ traits from a particular location) and the goal of the forecasting (e.g., short- vs. long-term) researchers and practitioners need to be equipped to switch between approaches lying in the spectrum from fully mechanistic trait-based (therefore complex) models, through simpler classical compartment and Anderson-May type models, to purely phenomenological and statistical modeling. For example simple first order auto-regressive linear models work surprisingly well for short term forecasting (Johansson et al., 2019; Li et al., 2019). But for forecasting over longer spatio-temporal scales, trait-based approaches will be needed. These approaches are particularly important in the face of ever-increasing underlying regime shifts in VBD dynamics as well as external controls on vector populations.

Efforts to develop hierarchical model validation methods for the types of complex trait-based dynamical models described here are ongoing (LaDeau et al., 2011; Johnson et al., 2013, 2014; Sun et al., 2015). These include Bayesian methods, which allow quantification of uncertainty and the incorporation of prior data (Clark, 2007; Hooten and Hobbs, 2015). Not only do the statistical methods for VBD dynamical systems need to be refined, but as they are developed it is important that these methods are made accessible for non-statisticians doing research in this area. This requires training a new generation of researchers in both the new modeling techniques so they can develop models that include details such as behavior, as well as statistical techniques appropriate for parameterizing and validating the models as they are developed. It is inevitable and useful that multiple models will be built to address the same question within any of the compartments of a trait-based framework (Johnson and Omland, 2004). For example, there are multiple ways to predict population growth from underlying life history traits (Amarasekare and Coutinho, 2013). Comparing the predictions from multiple models allows us to identify which models are most appropriate for a particular questions and systems. The VBD community can facilitate this critical step in creating useful models by making validation data sets and code used to generate models publicly available and accessible, and standardized metrics of goodness-of-fit or similar should be reported for all models against validation sets (Johansson et al., 2019). These steps would enable model comparison and multi-model ensembles to be used for future predictions.

## CONCLUSION

Building a fully trait-based approach to modeling VBD dynamics is not the “quick and easy path” (Kershner and Lucas, 1980). It is data-hungry and requires extensive efforts to build models that integrate knowledge about processes from the individual to populations and beyond. However, in comparison to phenomenological approaches (e.g., correlative or data mining approaches such as high-dimensional regression analyses) taking a more mechanistic approach, in general, provides a better way to extrapolate dynamics across time or space (Bayarri et al., 2009). Moreover, multiple modeling approaches should be compared (Shaw et al., 2019), and simulation and mathematical modeling approaches can be combined (Perkins et al., 2013). Mechanistic understanding and extrapolation are critical goals in light of the rapid rates of disease emergence and changes in climate and other environmental drivers, beyond regimes that have historically existed on Earth. By explicitly modeling the variation in a given trait and its effect on evolutionary fitness, population dynamics, and transmission, trait-based approaches could be used to incorporate trait evolution into transmission models. For instance, although we have focused on traits that directly affect vector population growth in idealized conditions, the addition of other traits that are mediated by human intervention, such as insecticide resistance, is also possible within this framework. The evolution of insecticide resistance is arguably the largest challenge to sustainable management of vector-borne diseases. A trait-based approach has the potential to better predict the implications of both current (e.g., chemical pesticides) and emerging control measures (e.g., genetically altered vectors) that inherently alter traits, while suggesting innovative and nuanced ways to apply control in a way that to anticipate changes driven by the inherent complexities of these systems.

## Supplementary Material

Supplementary Material
